# Potential Role of Aromatase over Estrogen Receptor Gene Polymorphisms in Migraine Susceptibility: A Case Control Study from North India

**DOI:** 10.1371/journal.pone.0034828

**Published:** 2012-04-12

**Authors:** Jayashri Ghosh, Gunjan Joshi, Sunil Pradhan, Balraj Mittal

**Affiliations:** 1 Department of Genetics, Sanjay Gandhi Post Graduate Institute of Medical Sciences (SGPGIMS), Lucknow, India; 2 Department of Neurology, Sanjay Gandhi Post Graduate Institute of Medical Sciences (SGPGIMS), Lucknow, India; California Pacific Medicial Center Research Institute, United States of America

## Abstract

**Background:**

The present study was undertaken to find out the role of estrogen pathway related gene polymorphisms in susceptibility to migraine in Northern Indian population. Aromatase, *CYP19A1* (rs10046 and rs4646); estrogen receptors, *ESR1* (rs2234693, rs1801132, rs2228480 and rs9340799) and *ESR2* (rs1271572 and rs1256049) polymorphisms were selected for the present study.

**Methodology/Principal Findings:**

The patients were recruited in two cohorts – primary (207) and replicative (127) along with 200 healthy controls and genotyped for various polymorphisms. Logistic regression analysis was applied for statistical analyses. The results were validated in the replicative cohort and pooled by meta analysis using Fisher's and Mantel-Haenszel test. Furthermore, Benjamini – Hochberg false discovery rate test was used to correct for multiple comparisons. *CYP19A1* rs10046 and *CYP19A1* rs4646 polymorphisms were found to confer risk and protective effect, respectively. Out of four *ESR1* polymorphisms, only rs2234693 variant allele was significantly associated in migraine with aura. No significant associations were observed for *ESR2* polymorphisms. Significant haplotypes were identified for *CYP19A1* and *ESR1* polymorphisms. Gene- gene interactions of genotypes as well as haplotypes were observed for *CYP19A1*- *ESR1* showing both risk and protective combinations.

**Conclusion:**

We strongly suggest *CYP19A1* polymorphisms to be the major contributing factors in migraine susceptibility instead of genetic variants of estrogen receptors.

## Introduction

Migraine is a debilitating neurological disorder affecting about 10% of world's population [1]. It can be broadly classified into two types- Migraine without aura (MO) and Migraine with aura (MA). It is a complex disorder involving interplay between genes and environmental factors. The exact pathophysiology of migraine is still unclear. However, the gender biasness in the incidence of migraine has paved the way for hormonal theory. Fluctuating hormone (especially estrogen) levels in women are considered to be the major contributing factors. It is observed that both estrogen withdrawal as well as high estrogen levels increase migraine risk in women [2]. Various genes of estrogen pathway are involved in estrogen signaling and its downstream effects. Hence, a detailed understanding of the polymorphisms and their functional effects may provide useful insights in the field of migraine pathophysiology.

*CYP19A1* (cytochrome P450, family 19, subfamily A, polypeptide 1) gene encodes aromatase enzyme which is involved in the final step of estrogen synthesis. Varying estrogen levels are associated with polymorphisms in the 3′ UTR (rs10046 C>T and rs4646 G>T) of this gene [3], [4]. However, there is a single study on role of rs10046 polymorphism in migraine [5].

Estrogen imparts its genomic effects through its receptors. The most common are the ligand gated receptors (ESRα encoded by *ESR1* gene and ESRβ encoded by *ESR2* gene). Many studies in migraine have focused on *ESR1* gene polymorphisms. Intronic polymorphisms [rs2234693 (*ESR1 Pvu*IIC>T) and rs9340799 (*ESR1 Xba*IA>G)] are associated with serum estradiol concentrations [6], [7]. The rs2234693 has been previously studied [8] whereas the other intronic polymorphism (rs9340799) has not yet been explored in migraine. Reports on synonymous exonic polymorphisms [rs1801132 (C325G) and rs2228480 (G594A)] of *ESR1*with migraine are conflicting [8], [9], [10], [11], [12]. Two of the above polymorphisms (rs2234693 and rs1801132) have also been studied by our group previously [13]. *ESR2* promoter polymorphism (rs1271572) may influence transcriptional factor binding and gene expression [14]. On the other hand, exonic polymorphism (rs1256049) results in a silent change which may affect mRNA folding, transcription and stability [15]. These polymorphisms have been found to be associated with comorbid diseases like cardiovascular disorder [15]. However, no group has studied these polymorphisms in migraine.

The present study was undertaken to explore the role of polymorphisms in genes of estrogen pathway in migraine susceptibility in Northern Indian population. The *CYP19A1* (rs10046 and rs4646), *ESR1* (rs2234693, rs1801132, rs2228480 and rs9340799) and *ESR2* (rs1271572 and rs1256049) polymorphisms were selected for the present study. The conflicting reports on *ESR1* gene polymorphisms and lack of reports on *CYP19A1* and *ESR2* gene polymorphisms led us to validate the results in a replicative cohort and finally pooling the results by meta analysis.

## Results

The genotypic and allelic frequencies of the study subjects are shown in Tables S1, S2, S3, S4, S5, S6, S7, and S8. All the studied polymorphisms followed Hardy Wienberg equilibrium in the control population.

### *CYP19A1* gene polymorphisms (rs10046 and rs4646)

#### *CYP19A1* rs10046 polymorphism

In the primary cohort, the frequencies of heterozygous (CT) and variant (TT) genotypes were significantly higher (p<0.001) in migraine patients as compared to healthy controls (HC). In case of CT, subgroup analysis showed significant association in migraine without aura (MO) only. Further subgroup analysis on the basis of gender also yielded statistically significant results in migraine and MO for both the genders. However, the results could be replicated in females only. Significant associations were observed for TT genotype in both migraines without and with aura (MA). Sub stratification on the basis of gender yielded significant results in females only. All the significant results were replicated. Similarly, we found statistically significant p values (p_corr_ = 0.01) on applying Fisher's method. Mantel – Haenszel test odds ratios confirmed the risk of heterozygous and variant genotypes in migraine susceptibility (Table 1).

**Table 1:** Association study of CYP19A1 rs10046 polymorphism.

	Primary cohort		Replicative cohort		Meta analysis			
					Fisher's method		Benjamini-Hochberg FDR test	Mantel-Haenszel test
**Association of CT genotype with migraine susceptibility**								
	**p** a	**OR(95%CI)**	**p** a	**OR(95%CI)**	**X^2^**	**p** a	**p_corr_** a	**OR_MH_(95%CI)**
Migraine Vs HC	**<0.0001**	2.937(1.893–4.556)	**<0.0001**	2.817(1.708–4.645)	46.66	**<0.0001**	**0.01**	1.925(1.431–2.59)
MO Vs HC	**<0.0001**	3.907(2.291–6.661)	**<0.0001**	3.103(1.785–5.397)	48.46	**<0.0001**	**0.01**	2.159(1.548–3.012)
Females								
Migraine Vs HC	**0.001**	2.560(1.507–4.349)	**0.00018**	3.165(1.732–5.784)	31.06	**<0.0001**	**0.01**	1.724(1.207–2.464)
MO Vs HC	**0.00027**	3.287(1.730–6.243)	**<0.0001**	3.385(1.740–6.587)	32.48	**<0.0001**	**0.01**	1.974(1.321–2.951)
**Association of TT genotype with migraine susceptibility**								
Migraine Vs HC	**<0.0001**	9.958(4.328–22.913)	**<0.0001**	6.687(2.637–16.961)	52.64	**<0.0001**	**0.01**	4.641(2.589–8.32)
MO Vs HC	**<0.0001**	13.134(5.301–32.542)	**0.0001**	3.103(1.785–5.397)	53.88	**<0.0001**	**0.01**	4.749(2.576–8.756)
MA Vs HC	**0.00018**	6.579(2.446–17.692)	**0.013**	6.052(1.467–24.970)	25.92	**<0.0001**	**0.01**	4.552(2.151–9.632)
Females								
Migraine Vs HC	**<0.0001**	14.681(4.806–44.846)	**0.00018**	10.314(3.043–34.952)	43.14	**<0.0001**	**0.01**	7.095(3.243–15.53)
MO Vs HC	**<0.0001**	16.278(4.892–54.170)	**0.00027**	10.712(2.983–38.464)	40.7	**<0.0001**	**0.01**	6.708(2.961–15.19)
MA Vs HC	**<0.0001**	13.065(3.782–45.130)	**0.012**	9.452(1.626–54.938)	24.12	**<0.0001**	**0.01**	8.135(3.192–20.74)
**Association of T allele with migraine susceptibility**								
Migraine Vs HC	**<0.0001**	2.618(1.943–3.529)	**<0.0001**	2.248(1.603–3.152)	69.78	**<0.0001**	**0.01**	2.487(1.991–3.106)
MO Vs HC	**<0.0001**	2.942(2.107–4.109)	**<0.0001**	2.314(1.610–3.327)	68.38	**<0.0001**	**0.01**	2.662(2.085–3.398)
MA Vs HC	**0.0002**	2.116(1.425–3.141)	**0.018**	2.030(1.131–3.645)	25.08	**<0.0001**	**0.01**	2.166(1.568–2.993)
Females								
Migraine Vs HC	**<0.0001**	2.810(1.950–4.047)	**<0.0001**	2.479(1.655–3.713)	57.56	**<0.0001**	**0.01**	2.659(2.03–3.483)
MO Vs HC	**<0.0001**	2.977(1.969–4.500)	**<0.0001**	2.533(1.643–3.905)	51.7	**<0.0001**	**0.01**	2.749(2.041–3.702)
MA Vs HC	**<0.0001**	2.584(1.622–4.115)	**0.016**	2.303(1.168–4.541)	27.6	**<0.0001**	**0.01**	2.505(1.712–3.666)
**Association using dominant (CT+TT) model**								
Migraine Vs HC	**<0.0001**	3.557(2.326–5.439)	**<0.0001**	3.147(1.934–5.121)	63.26	**<0.0001**	**0.01**	3.419(2.49–4.693)
MO Vs HC	**<0.0001**	4.713(2.807–7.914)	**<0.0001**	3.442(2.007–5.903)	62.12	**<0.0001**	**0.01**	4.046(2.799–5.849)
MA Vs HC	**0.003**	2.363(1.349–4.136)	**0.045**	2.394(1.021–5.616)	17.82	**0.0013**	**0.01**	2.486(1.568–3.94)
Females								
Migraine Vs HC	**<0.0001**	3.372(2.023–5.621)	**<0.0001**	3.625(2.012–6.534)	47.2	**<0.0001**	**0.01**	3.436(2.345–5.034)
MO Vs HC	**<0.0001**	4.173(2.241–7.769)	**<0.0001**	3.868(2.018–7.412)	43.8	**<0.0001**	**0.01**	3.931(2.522–6.128)
MA Vs HC	**0.005**	2.569(1.322–4.989)	**0.037**	2.967(1.069–8.236)	17.2	**0.0018**	**0.01**	2.672(1.544–4.623)

MA migraine with aura, MO migraine without aura, HC healthy controls, OR odds ratio, CI confidence interval, OR_MH_ Mantel – Heanszel odds ratio.

a p Values in bold denotes significance.

At allelic level, significant results were seen with migraine as well as subgroups in the primary cohort except male MA (data not shown). All the results were replicated except for males. On pooling the data, significance was retained in all these subgroups (p_corr_ = 0.01) showing risk of variant allele. Carrier analysis using dominant model showed highly significant results with migraine and its subgroups. However significance could not be obtained in male patients (Table 1).

#### *CYP19A1* rs4646 polymorphism

In the primary cohort, the heterozygous (GT) genotype did not show significant associations with migraine or its clinical subgroups (data not shown). However, on gender stratification, female migraine patients (p = 0.022; OR = 0.544) and female MA patients (p = 0.015; OR = 0.427) showed protective effect. We were able to replicate the results. On pooling, significant results were obtained in both the subgroups showing an overall protective effect of the genotype (Table 2).

**Table 2:** Association study of CYP19A1 rs4646 polymorphism.

	Primary cohort		Replicative cohort		Meta analysis			
					Fisher's method		Benjamini-Hochberg FDR test	Mantel-Haenszel test
	p^a^	OR(95%CI)	p^a^	OR(95%CI)	X^2^	p^a^	p_corr_ a	OR_MH_(95%CI)
**Association of GT genotype with migraine susceptibility**								
Females								
Migraine Vs HC	**0.022**	0.544(0.324–0.915)	**0.002**	0.388(0.212–0.711)	20.06	**0.0005**	**0.01**	0.5465(0.3781–0.7899)
MA Vs HC	**0.015**	0.427(0.215–0.846)	**0.029**	0.296(0.099–0.880)	15.48	**0.0038**	**0.01**	0.4856(0.2789–0.8456)
	**Association of T allele with migraine susceptibility**							
MA Vs HC	**0.034**	0.639(0.422–0.966)	**0.014**	0.417(0.208–0.835)	15.3	**0.0041**	**0.01**	0.5695(0.4007–0.8093)
Females								
Migraine Vs HC	**0.011**	0.629(0.441–0.897)	**0.029**	0.641(0.430–0.956)	16.1	**0.0029**	**0.01**	0.6353(0.4871–0.8287)
MA Vs HC	**0.002**	0.452(0.274–0.747)	**0.027**	0.416(0.191–0.906)	19.64	**0.0006**	**0.01**	0.442(0.29–0.6737)
**Association using dominant (GT+TT) model**								
Females								
Migraine Vs HC	**0.010**	0.529(0.326–0.857)	**0.004**	0.455(0.265–0.780)	20.26	**0.0004**	**0.01**	0.4953(0.3457–0.7096)
MA Vs HC	**0.003**	0.380(0.201–0.721)	**0.016**	0.303(0.114–0.801)	19.9	**0.0005**	**0.01**	0.3531(0.2072–0.6017)

MA migraine with aura, HC healthy controls, OR odds ratio, CI confidence interval, OR_MH_ Mantel – Heanszel odds ratio.

a p Values in bold denotes significance.

At allelic level, protective effect of the variant T allele was evident in MA patients in both the cohorts. Similar associations were observed for female migraine and female MA patients. Furthermore, we found highly significant associations on pooling. In addition, dominant model also showed significant results for female migraine patients and female MA patients in both the cohorts as well as on pooling (Table 2).

#### *CYP19A1*: LD and Haplotype Analysis

On LD analysis, D′ value of 0.4637 was observed, suggesting a moderate linkage disequilibrium. The frequency of rs10046T-rs4646G haplotype was significantly higher in patients of both primary (37.7%) and replicative (33.1%) cohorts as compared to HC (20.0%), imparting significant results. Similar results were obtained for the rs10046T-rs4646T haplotype. The pooling of data validated these results (Table 3). On subgrouping, similar results were observed for MO. However, we did not find significant predominance of any haplotype groups in MA. Furthermore, stratification on the basis of gender again yielded significant results for rs10046T-rs4646G haplotype. No significant associations were observed for other haplotype groups. All the significant observations were replicated and validated (Table 3).We did not find any association in male subgroups (data not shown).

**Table 3:** Haplotype analysis of CYP19A1 gene polymorphisms.

Haplotype^a^(rs10046-rs4646)	Healthy Controls	Primary cohort			Replicative cohort			Meta Analysis			
								Fisher's method		Benjamini-Hochberg FDR test	Mantel-Haenszel test
	N(%)	N(%)	p^b^	OR(95%CI)	N(%)	p^b^	OR(95%CI)	X^2^	p^b^	p_corr_ b	OR_MH_(95%CI)
**Migraine Vs HC**											
00	88(44.0)	67(32.4)	Reference		47(37.0)	Reference		-	-	**-**	-
01	62(31.0)	43(20.8)	0.716	0.911(0.551–1.505)	25(19.7)	0.345	0.755(0.421–1.354)	-	-	**-**	-
10	40(20.0)	78(37.7)	**0.0002**	2.561(1.559–4.207)	42(33.1)	**0.018**	1.966(1.124–3.440)	25.08	**<0.0001**	**0.01**	2.219(1.589–3.1)
11	10(5.0)	19(9.2)	**0.031**	2.496(1.089–5.718)	13(10.2)	**0.052**	2.434(0.992–5.970)	12.86	**0.0120**	**0.02**	2.026(1.132–3.628)
**MO Vs HC**											
00	88(44.0)	37(28.7)	Reference		35(35.4)	Reference		-	-		-
01	62(31.0)	28(21.7)	0.812	1.074(0.596–1.935)	21(21.2)	0.618	0.852(0.453–1.601)	-	-		-
10	40(20.0)	51(39.5)	**<0.0001**	3.032(1.724–5.334)	32(32.3)	**0.024**	2.011(1.095–3.694)	25.52	**<0.0001**	**0.01**	2.273(1.577–3.275)
11	10(5.0)	13(10.1)	**0.015**	3.092(1.245–7.677)	11(11.1)	**0.034**	2.766(1.079–7.092)	15.16	**0.0044**	**0.01**	1.949(1.053–3.606)
**MA Vs HC**											
00	88(44.0)	30(38.5)	Reference		12(42.9)	Reference		-	-	-	-
01	62(31.0)	15(35.9)	0.337	0.710(0.352–1.429)	4(14.3)	0.213	0.473(0.146–1.536)	-	-	**-**	-
10	40(20.0)	27(34.6)	**0.037**	1.980(1.044–3.756)	10(35.7)	0.196	1.833(0.732–4.594)	-	-	**-**	-
11	10(5.0)	6(7.7)	0.311	1.760(0.590–5.254)	2(7.1)	0.646	1.467(0.286–7.513)	-	-	**-**	-
**Female Migraine Vs Female HC**											
00	57(42.9)	45(31.9)	Reference		31(33.3)	Reference		-	-	**-**	-
01	44(33.1)	29(20.6)	0.562	0.835(0.453–1.537)	21(22.6)	0.706	0.878(0.445–1.731)	-	-	**-**	-
10	24(18.0)	55(39.0)	**0.001**	2.903(1.564–5.389)	32(34.4)	**0.010**	2.452(1.234–4.871)	23.03	**0.0001**	**0.01**	2.663(1.764–4.021)
11	8(6.0)	12(8.5)	0.198	1.900(0.716–5.044)	9(9.7)	0.174	2.069(0.725–5.899)	-	-		-

MA migraine with aura, MO migraine without aura, HC healthy controls, OR odds ratio, CI confidence interval, OR_MH_ Mantel – Heanszel odds ratio.

a 0&1 indicates wild and variant, respectively.

b p Values in bold denotes significance.

### *ESR1* gene polymorphisms (rs2234693, rs1801132, rs2228480 and rs9340799)

Four polymorphisms of *ESR1* gene were explored for association with migraine and its clinical subgroups. In rs2234693, the variant allele (T) showed a borderline significance in MA patients in the primary cohort. Significant differences were observed in the replicative cohort. The risk of the variant allele was evident on data pooling (Table 4). No significant differences in genotypic and allelic distribution of rs1801132, rs2228480 and rs9340799 polymorphisms were observed among migraine patients and HC in both the cohorts. On sub-classification of migraine patients into MO and MA, the genotypic and allelic distributions were found to be insignificant when compared to HC. Further stratification of data on gender basis also did not yield any significant results (Table 4).

**Table 4:** Association study of ESR1 polymorphisms.

	Primary cohort		Replicative cohort		Meta analysis			
					Fisher's method		Benjamini-Hochberg FDR test	Mantel-Haenszel test
	p^a^	OR(95%CI)	p^a^	OR(95%CI)	X^2^	p^a^	p_corr_ a	OR_MH_(95%CI)
**ESR1 rs2234693**								
**Association of TT genotype with migraine susceptibility**								
Migraine Vs HC	**0.007**	2.477(1.275–4.813)	0.081	1.847(0.927–3.681)	-	-	-	-
MO Vs HC	**0.006**	2.822(1.350–5.898)	0.498	1.302(0.607–2.792)	-	-	-	-
MA Vs HC	0.177	1.911(0.747–4.891)	**0.002**	6.205(1.945–19.792)	-	-	-	-
**Association of T allele with migraine susceptibility**								
Migraine Vs HC	**0.002**	1.547(1.167–2.050)	0.237	1.217(0.879–1.684)	-	-	-	-
MO Vs HC	**0.003**	1.612(1.172–2.219)	0.112	0.737(0.506–1.074)	-	-	-	-
MA Vs HC	0.060	1.438(0.985–2.100)	**0.004**	2.305(1.305–4.070)	16.66	**0.0023**	**0.01**	1.634(1.196–2.233)
**ESR1 rs1801132**								
**Association of GG genotype with migraine susceptibility**								
Migraine Vs HC	0.138	1.705(0.843–3.450)	0.431	0.701(0.289–1.698)	-	-	-	-
MO Vs HC	0.128	1.842(0.839–4.046)	0.486	0.710(0.271–1.860)	-	-	-	-
MA Vs HC	0.399	1.491(0.590–3.767)	0.595	0.652(0.135–3.152)	-	-	-	-
**ESR1 rs2228480**								
**Association of AA genotype with migraine susceptibility**								
Migraine Vs HC	0.420	0.657(0.238–1.820)	0.811	0.870(0.279–2.719)	-	-	-	-
MO Vs HC	0.415	0.603(0.179–2.030)	0.829	0.874(0.256–2.980)	-	-	-	-
MA Vs HC	0.662	0.740(0.191–2.860)	0.933	0.911(0.106–7.860)	-	-	-	-
**ESR1 rs 9340799**								
**Association of GG genotype with migraine susceptibility**								
Migraine Vs HC	0.883	1.047(0.566–1.940)	0.878	1.055(0.531–2.094)	-	-	-	-
MO Vs HC	0.513	1.254(0.636–2.475)	0.288	0.647(0.290–1.444)	-	-	-	-
MA Vs HC	0.455	0.708(0.286–1.751)	**0.016**	4.000(1.296–12.342)	-	-	-	-

MA migraine with aura, MO migraine without aura, HC healthy controls, OR odds ratio, CI confidence interval, OR_MH_ Mantel – Heanszel odds ratio.

a p Values in bold denotes significance.

#### *ESR1*: LD and Haplotype Analysis

ESR1 rs2234693 and rs9340799 were in strong linkage disequilibrium (D′ = 0.6222). No other pairs of polymorphisms were found to be in linkage disequilibrium (D′<0.5). Haplotypes were constructed for the selected polymorphisms (Table S9). The haplotype comprising of the entire wild ones (A) was taken as reference. In the primary cohort, frequency of one of the haplotype group (B) was found to be significantly lower in patients as compared to controls. The result was replicated and pooling also confirmed the association (p_corr_ = 0.01) showing a protective effect (OR = 0.129) (Table S9).

### *ESR2* gene polymorphisms (rs1271572 and rs1256049)

No significant associations were observed for rs1271572 and rs1256049 at genotypic and allelic levels. No significant differences were evident on subgroup analysis (Table S10). Furthermore, these two polymorphisms were not in linkage disequilibrium (D′ = 0.3649).

### Gene- gene interactions

The *CYP19A1* rs10046 polymorphism was found to have significant interactions with both *ESR1* and *ESR2* gene polymorphisms. Significant interactions were observed for *CYP19A1* rs10046 with *ESR2* rs1271572, *ESR1* rs2234693 and *ESR1* rs9340799 polymorphisms conferring risk for migraine susceptibility. Combinations of *ESR2* rs1271572 AC genotype with both heterozygous as well as variant genotypes of *CYP19A1* rs10046 were significantly higher in patients as compared to controls. These interactions were replicated and validated on pooled analysis (Table 5). However, the degree of association was not increased on interaction than the one observed in case of individual (rs10046) polymorphism.

**Table 5:** Gene-Gene Interaction of genotypes.

Genotype interactions^a^	Primary cohort		Replicative cohort		Meta analysis			
					Fisher's method		Benjamini-Hochberg FDR test	Mantel-Haenszel test
	p^b^	OR(95%CI)	p^b^	OR(95%CI)	X^2^	p^b^	p_corr_ b	OR_MH_(95%CI)
**CYP19A1 rs10046- ESR2 rs1271572**								
1-1	**0.034**	1.676(1.038–2.706)	**0.015**	1.938(1.138–3.301)	15.16	**0.0044**	**0.01**	1.637(1.155–2.32)
2-1	**0.021**	4.584(1.264–16.620)	**0.019**	5.068(1.299–19.778)	15.65	**0.0035**	**0.01**	4.202(1.657–10.66)
**CYP19A1rs10046-ESR1 rs2234693**								
2-1	**<0.0001**	12.535(3.666–42.856)	**0.027**	4.646(1.190–18.134)	26.84	**<0.0001**	**0.01**	8.138(3.385–19.56)
1-2	**0.025**	2.662(1.130–6.269)	**0.012**	3.058(1.280–7.307)	16.22	**0.0027**	**0.01**	2.053(1.131–3.727)
**CYP19A1rs10046- ESR1 rs9340799**								
2-1	**0.001**	12.970(2.960–56.836)	**0.014**	7.238(1.490–35.162)	22.36	**0.0002**	**0.01**	7.91(2.715–23.04)
1-2	0.054	2.400(0.985–5.848)	**0.014**	3.187(1.269–8.003)	14.38	**0.0062**	**0.01**	2.081(1.11–3.901)
**CYP19A1 rs4646-ESR1 rs9340799**								
1-1	**0.017**	0.513(0.297–0.887)	**0.006**	0.387(0.198–0.757)	18.38	**0.001**	**0.01**	0.3457(0.2221–0.5382)

OR odds ratio, CI confidence interval, OR_MH_ Mantel – Heanszel odds ratio.

a 1&2 represent heterozygous and variant genotypes, respectively.

b p Values in bold denotes significance.

The SNPs of *CYP19A1*-*ESR1* also interacted significantly. Combinations with two of the *ESR1* polymorphisms (rs2234693 and rs9340799) and *CYP19A1* rs10046 were significantly higher in patients. In these polymorphisms, each of the heterozygous – variant genotype combination was found to be significantly associated with migraine. The associations were replicated and validated showing significantly higher risk for patients having these genotype combinations. Furthermore, the risk was increased in all these interactions than observed for individual polymorphisms (Table 5).

The *CYP19A1* rs4646 polymorphism was found to have significant interactions with only *ESR1* rs9340799 polymorphism. The combination of heterozygous genotypes of these polymorphisms conferred a significant (p_corr_ = 0.01) protective effect (OR_MH_ = 0.3457) (Table 5). No significant associations were observed with other *ESR1* or *ESR2* polymorphisms (data not shown).

### Gene- gene interactions of haplotypes

The *CYP19A1* and *ESR1* haplotypes were analyzed for interactions. Interactions between a single haplotype combination (*CYP19A1* 01-*ESR1* H) was significantly associated and another (*CYP19A1* 10-*ESR1* H) combination and showed borderline association in the primary cohort. However, only one of the interactions (*CYP19A1* 01-*ESR1* H) was replicated. Furthermore, on pooling both these interactions (*CYP19A1* 01-*ESR1* H and *CYP19A1* 10-*ESR1* H) were found to have significant protective effect and risk respectively (Table 6).

**Table 6:** Gene-Gene Interaction of haplotypes.

Haplotype interactions	Primary cohort		Replicative cohort		Meta analysis			
					Fisher's method		Benjamini-Hochberg FDR test	Mantel-Haenszel test
	p^a^	OR(95%CI)	p^a^	OR(95%CI)	X^2^	p^a^	p_corr_ a	OR_MH_(95%CI)
**CYP19A1 – ESR1**								
01-H	**0.025**	0.272(0.087–0.851)	**0.021**	0.089(0.012–0.691)	15.10	**0.0045**	**0.01**	0.153(0.058–0.406)
10-H	**0.032**	2.894(1.093–7.663)	0.091	2.453(0.866–6.948)	11.68	**0.0199**	**0.02**	2.555(1.271–5.138)

OR odds ratio, CI confidence interval, OR_MH_ Mantel – Heanszel odds ratio.

a p Values in bold denotes significance.

## Discussion

Migraine is predominant in women as compared to men (3∶1) [16]. The role of female hormones especially estrogen cannot be overlooked in this gender biasness. Hence, studies have focused on the relationship between estrogen and migraine. However, studies in genetic polymorphisms in estrogen pathway have been limited to *ESR1* SNPs. Therefore, we have also included less studied but functionally relevant *CYP19A1* and *ESR2* gene polymorphisms in our study.

*CYP19A1* is located in short arm of chromosome 15 (15q21). It is a member of cytochrome P450 family and codes for aromatase enzyme which catalyzes the final step of estrogen biosynthesis. Our data indicates strong association of *CYP19A1* 3′UTR polymorphisms with migraine susceptibility. The variant genotype and allele of rs10046 polymorphism impart a potentially significant migraine risk especially in females. The carrier analysis further implies the effect of variant allele in dominant form as well. Dunning *et al.* [17] for the first time reported the association of C allele with low estrone and estradiol levels. The result was replicated by Haiman *et al.* [3] This marker has been associated with altered pituitary suppression in premenopausal women [18]. It also showed significant risk with breast cancer [19] and essential hypertension [20]. On the contrary, rs4646 variant (T) polymorphism was found to have a protective effect in susceptibility to migraine. Haiman *et al.* [3] reported significantly increased estrone and estradiol levels in individuals with GG genotype. This polymorphism has a significant role in determining efficacy of antiaromatase therapy [21]. The 3′UTR polymorphisms are located 142 bp apart. As expected, these polymorphisms are in linkage disequilibrium. Haplotype analysis of *CYP19A1* polymorphisms revealed the significant predominance of two haplotype groups in migraine patients. Both these haplotype groups consisted of the variant of rs10046 polymorphism, reconfirming its role in migraine susceptibility.

Estrogen hormone carries out its physiological functions through its receptors. ESRα is expressed by many of the trigeminal neurons. The gene encoding estrogen receptor alpha, (*ESR1*) localized in 6q25.1 locus is the most widely studied hormonal pathway gene in migraine. We analyzed two intronic (intron 1) and two exonic (exons 4 & 8) polymorphisms of this gene. However, we could establish a significant relationship of a single intronic polymorphism (rs2234693) in migraine with aura patients. The existing literature reveals conflicting reports [8], [13]. The nearby SNP rs9340799 was not associated with migraine. The lack of association of the exonic polymorphisms is previously documented by several authors [8], [9]. These polymorphisms do not result in any change in amino acid sequence. This further strengthens our findings.

On the contrary, a recent meta analysis of *ESR1* polymorphisms reported significant associations of exonic polymorphisms [22]. However, among six studies [5], [8], [9], [10], [11], [13] on *ESR1* rs1801132 polymorphism, only two studies [5], [11] reported significant associations with migraine. Similarly, only a single study [12] among four reports on *ESR1* rs2228480 polymorphism showed significant findings [9], [10], [11], [12]. Thus, our results are in accordance with majority of the published results. The discordance with the results of meta analysis could be explained by the large variations in sample size of the included studies, which could tilt the final outcome on the studies with larger samples.

The intronic polymorphisms are at a distance of 50 bp and hence are in strong linkage disequilibrium. The polymorphisms were not in LD with other polymorphisms. Similarly, Colson *et al.* [8] did not find LD between exon 8 marker with intronic and exon 4 markers. However, the authors reported LD between intron1 and exon 4 markers. But the distance (∼102 kb) between the markers could explain our findings.

We were able to identify a particular haplotype group showing protective effect of migraine. This particular group consists of variant allele (G) of one intronic polymorphism (rs9340799) whereas the wild alleles of other polymorphisms. Hence, it shows the antagonistic role of the two intronic polymorphisms. Literature review also reveals that *Xba*I*G (variant) and *Pvu*II*C (wild) SNPs are associated with elevated serum estradiol (E2) production [6], [7]. Hence, these alleles may impart a protective effect. The results of our study could be justified by the role of amino terminal portion in transcriptional activation and its association with various pathological conditions [23]. In other words, we report that the effect of *ESR1* polymorphisms on migraine risk diminish from amino to carboxy terminal.

ESRβ is mostly expressed by inhibitory neurons in cerebral cortex. Therefore, it regulates cortical excitability and spreading depression [24] involved in migraine pathophysiology. It is encoded by *ESR2* gene located in 14q22-24. A promoter polymorphism (rs1271572) has a functional effect on transcriptional factor binding and gene expression [14]. The other selected polymorphism (rs1256049) is present in exon 5 which results in a silent change in amino acid. However, we were unable to find an association of these polymorphisms in migraine. No previous reports are available for these polymorphisms in migraine.

We also studied the genetic interactions. Our study supports the significant interactions between *CYP19A1* and *ESR* polymorphisms. Thus we infer that though individual polymorphisms have no role but in the presence of other genetic variants, these could be a possible risk factor (as in case of *ESR1* intronic polymorphisms). We obtained antagonistic interaction of *ESR1* rs9340799 with the *CYP19A1* 3′UTR polymorphisms. This SNP showed an increased risk with rs10046 polymorphism whereas a protective effect with rs4646 polymorphism. It implies the role of *CYP19A1* polymorphisms in defining the effect on migraine susceptibility. Similarly, we also analyzed gene – gene interaction of haplotypes. In this case also antagonistic role of *ESR1* H haplotype was observed with *CYP19A1* haplotypes. These findings further strengthen the effect of *CYP19A1* polymorphisms on migraine susceptibility. This is further supported by the fact that haplotype H consists of both the variants of intronic polymorphisms shown to have opposite effect on migraine susceptibility. So the individual effect of the haplotype is more or less nullified. In other words, the same haplotype of *ESR1* may show protective effect or risk depending on the *CYP19A*1 haplotypes. Hence, we conclude that *CYP19A1* polymorphisms and haplotypes are the major causal factors for migraine susceptibility. However, their effect is enhanced on genetic interaction with *ESR1* polymorphisms and haplotypes. No other group has analyzed or reported such genetic interactions.

The variants of associated polymorphisms (rs10046, rs4646 and rs2234693) are found to affect estrogen levels (either high or low). So, the bimodal effect of estrogen is implicated in migraine pathophysiology. Estrogen withdrawal is the most well accepted theory especially in migraine without aura. It is attributed to the fact that frequency of attacks increase with menstruation and use of oral contraceptives. In addition, migraine attacks subside during pregnancy and with menopause - two phases of high estrogen levels in women. On the other hand, high estrogen levels influence gene expression and signaling, finally leading to inflammatory pain [25]. Estrogen enhances cortical spreading depression involved in the pathophysiology of migraine with aura [26]. Hence abrupt estrogen decline as well as abnormally high estrogen levels leads to trigeminal pain [27].

Our study reports various significant findings. First of all, we are the first group to report significant associations of *CYP19A1* 3′ UTR polymorphisms. Secondly, we identified protective haplotype groups in *ESR1* gene. Thirdly, we defined significant genetic interactions between *CYP19A1*-*ESR1* gene polymorphisms. Fourthly, we were also able to show significant genetic interaction of haplotypes. Finally, we were able to replicate all the significant findings. Thus our results do not lack validation as is the case of other association studies. In addition, the data of both the cohorts were pooled using statistical tests (Fisher's method and Mantel- Haenszel test). So, in contrast to direct pooling, there is no need of additional adjustments or corrections. We are the first group to use such tests for validating data in defining role of hormone related genes in migraine.

In the era of genome wide association studies (GWAS), our candidate gene case-control study suffers from limitations especially in terms of sample size although our study achieved 80% power. Furthermore, limited sample size in some subgroups is another important issue. However, we have tried to overcome it by applying Benjamini – Hochberg FDR test for multiple comparisons. Secondly, wide confidence interval in case of rs10046 polymorphism (wild Vs variant genotype) is also of concern. In such cases, pooling by meta analysis has resulted in narrowing the confidence interval to some extent. Lastly, even though association studies are considered to be somewhat outdated in the GWAS era, our study provides some definite insights for future work.

In conclusion, we report the significant association of *CYP19A1* 3′UTR and *ESR1* rs2234693 polymorphism with migraine risk. We also found increased risk with certain *CYP19A1* and *ESR1* haplotype groups. Similarly, significant gene interactions of genotypes and haplotypes were also identified. Based on these findings, we suggest *CYP19A1* polymorphisms to be the major contributing factor in migraine susceptibility rather than *ESR1*and *ESR2* polymorphisms as shown by interactions of genotypes and haplotypes. In future, more studies in other ethnic groups should focus on *CYP19A1* polymorphisms to better understand the pathobiology of migraine.

## Materials and Methods

### Ethics statement

The study protocol was approved by the Institutional ethical committee of SGPGIMS Lucknow (India). Written informed consent was taken by all the participants.

### Subjects

Normotensive migraine patients were recruited from the Neurology Out Patient Clinic (OPD) of Sanjay Gandhi Postgraduate Institute of Medical Sciences (SGPGIMS), Lucknow, India. Diagnosis of migraine was carried out according to the criteria of the International Headache Society (IHS) [28]. The patients were recruited in two cohorts - primary and replicative. The patients (n = 207) recruited in the period 2006–2008 were included in primary cohort and those recruited from 2009 onwards (n = 127) were included in replicative cohort. Simultaneously, 200 healthy controls (HC) from volunteers like healthy staff members and general population were included in the study. The healthy controls were mean age, sex, ethnicity matched and free from any organic disease. The controls were especially checked for the presence of migraine and other headache disorders. Persons free from such headaches were enrolled for the study. The recruitment of the subjects was based on the inclusion and exclusion criteria [28]. In general, subjects with age of onset <50 years and attack frequency of at least 1 per month for previous 3 months were included. Migraine patients with comorbid disorders – vascular diseases (eg. hypertension, ischemic heart disease); neurological disorders (eg. epilepsy, stroke); hormonal disorders (eg. hypothyroidism) and non migrainous headaches (eg. tension headaches) were excluded. Secondary causes of migraine like post head injury migraineurs were also excluded from the study. The family history and general questionnaire of all the subjects recruited for the study were taken to ensure their ethnicity. After informed consent, 3 ml of blood was collected in EDTA vial and stored at −70°C.

### Methods

#### DNA isolation

Genomic DNA was extracted from frozen blood using the standard salting out method [29].

#### Genotyping of CYP19A1 polymorphisms

Genotyping of rs10046 and rs4646 polymorphisms were done by Hexaprimer Amplification Refractory Mutation System – polymerase chain reaction (PCR) as previously described [30].

#### Genotyping of ESR1 polymorphisms

Genotyping was done by PCR- restriction fragment length polymorphism (RFLP). The promoter polymorphisms were genotyped as previously described [31]. *ESR1* G594A was genotyped using *Btg*I [12] and C325G polymorphism was genotyped using *Hinf*I as described by Iwase et al. [32].

#### Genotyping of ESR2 polymorphisms

rs 1271572 polymorphism was amplified using self designed primers: 5′ CTGCCCACCCCTCTTCTC3′ and 5′ CCATCTTTGGAGCCTGTCTT3′ and genotyped by *Bsa*I whereas rs1256049 was genotyped using *Rsa*I as previously described [33].

#### Quality control

20% of samples were re-genotyped by another laboratory member to improve the quality of genotyping and its validity, and no discrepancy in genotyping was found. Genotyping of 5% of these samples was reconfirmed through sequencing.

#### Statistical analysis

Sample size was calculated using Quanto version 1.1.1.The study achieved 80% power. Goodness of fit χ^2^ test was used to check Hardy Weinberg Equilibrium in control population. Logistic regression analyses were done using SPSS version 15. The results were analyzed separately at genotypic and the allelic levels. Furthermore, linkage disequilibrium and haplotype analysis was done using SNP Analyzer version 1.2. In addition, the results were also analyzed using dominant model and trend per copy of haplotypes. Finally, gene-gene interactions were also analyzed.

The results of primary cohort were validated in the replicative cohort. On obtaining significant associations in both the cohorts, the results were pooled by meta analysis using Fisher's method (X^2^ and p value) and Mantel-Haenszel test using Open Epi version 2.3.1 for odds ratio (OR) calculation. Benjamini – Hochberg false discovery rate (FDR) test was used to correct for multiple comparisons yielding p_corr_. p_corr_ value less than 0.05 was considered as significant.

## Supporting Information

Table S1 **Genotypic and allelic distribution of** ***CYP19A1*** **rs10046 polymorphism in studied subjects.**
(DOC)

Table S2 **Genotypic and allelic distribution of** ***CYP19A1*** **rs4646 polymorphism in studied subjects.**
(DOC)

Table S3 **Genotypic and allelic distribution of** ***ESR1*** **rs2234693 polymorphism in studied subjects.**
(DOC)

Table S4 **Genotypic and allelic distribution of** ***ESR1*** **rs1801132 polymorphism in studied subjects.**
(DOC)

Table S5 **Genotypic and allelic distribution of** ***ESR1*** **rs2228480 polymorphism in studied subjects.**
(DOC)

Table S6 **Genotypic and allelic distribution of** ***ESR1*** **rs9340799 polymorphism in studied subjects.**
(DOC)

Table S7 **Genotypic and allelic distribution of** ***ESR2*** **rs1271572 polymorphism in studied subjects.**
(DOC)

Table S8 **Genotypic and allelic distribution of** ***ESR2*** **rs1256049 polymorphism in studied subjects.**
(DOC)

Table S9 **Haplotype analysis of** ***ESR1*** **gene polymorphisms.**
(DOC)

Table S10 **Association study of** ***ESR2*** **polymorphisms.**
(DOC)
