# Supplementary material for: Potential Role of Aromatase over Estrogen Receptor Gene Polymorphisms in Migraine Susceptibility: A Case Control Study from North India
Source: PLoS One. 2012 Apr 12;7(4):e34828. doi: 10.1371/journal.pone.0034828 (PMC3325278; doi:10.1371/journal.pone.0034828)
Supplement: Table S1 — Genotypic and allelic distribution of CYP19A1 rs10046 polymorphism in studied subjects. (DOC) [file pone.0034828.s001.doc]

**Table S 1: Genotypic and allelic distribution of *CYP19A1* rs10046 polymorphism in studied subjects**

|  | Genotypic distribution N(%) | | | Allelic distribution N(%) | |
| --- | --- | --- | --- | --- | --- |
|  | CC | CT | TT | C | T |
| Primary cohort | | | | | |
| Migraine(207) | 51(24.6) | 118(57.0) | 38(18.4) | 220(53.14) | 194(46.86) |
| MO(129) | 26(20.2) | 78(60.5) | 25(19.4) | 130(50.39) | 128(49.61) |
| MA(78) | 25(32.1) | 40(51.3) | 13(16.7) | 90(57.69) | 66(42.31) |
| Females |  |  |  |  |  |
| Migraine(141) | 37(26.2) | 74(52.5) | 30(21.3) | 148(52.48) | 134(47.52) |
| MO(84) | 19(22.6) | 48(57.1) | 17(20.2) | 86(51.19) | 82(48.81) |
| MA(57) | 18(31.6) | 26(45.6) | 13(22.8) | 62(54.39) | 52(45.61) |
| Males |  |  |  |  |  |
| Migraine(66) | 14(21.2) | 44(66.7) | 8(12.1) | 72(54.55) | 60(45.45) |
| MO(45) | 7(15.6) | 30(66.7) | 8(17.8) | 44(48.89) | 46(51.11) |
| MA(21) | 7(33.3) | 14(66.7) | 0(0) | 28(66.67) | 14(33.33) |
| Replicative cohort | | | | | |
| Migraine(127) | 34(26.8) | 76(59.8) | 17(13.4) | 144(56.69) | 110(43.31) |
| MO(99) | 25(25.3) | 61(61.6) | 13(13.1) | 111(56.06) | 87(43.94) |
| MA(28) | 9(32.1) | 15(53.6) | 4(14.3) | 33(58.93) | 23(41.07) |
| Females | | | | | |
| Migraine(93) | 23(24.7) | 57(61.3) | 13(14.0) | 103(55.38) | 83(44.62) |
| MO(72) | 17(23.6) | 45(62.5) | 10(13.9) | 79(54.86) | 65(45.14) |
| MA(21) | 6(28.6) | 12(57.1) | 3(14.3) | 24(57.14) | 18(42.86) |
| Males |  |  |  |  |  |
| Migraine(34) | 11(32.4) | 19(55.9) | 4(11.8) | 41(60.29) | 27(39.71) |
| MO(27) | 8(29.6) | 16(59.3) | 3(11.1) | 32(59.26) | 22(40.74) |
| MA(7) | 3(42.9) | 3(42.9) | 1(14.3) | 9(64.29) | 5(35.71) |
| Healthy controls | | | | | |
| HC(200) | 108(54.0) | 84(42.0) | 8(4.0) | 300(75.00) | 100(25.00) |
| Females(133) | 72(54.1) | 57(42.9) | 4(3.0) | 201(75.56) | 65(24.44) |
| Males(67) | 36(53.7) | 27(40.3) | 4(6.0) | 99(73.88) | 35(26.12) |
