# Supplementary material for: Potential Role of Aromatase over Estrogen Receptor Gene Polymorphisms in Migraine Susceptibility: A Case Control Study from North India
Source: PLoS One. 2012 Apr 12;7(4):e34828. doi: 10.1371/journal.pone.0034828 (PMC3325278; doi:10.1371/journal.pone.0034828)
Supplement: Table S2 — Genotypic and allelic distribution of CYP19A1 rs4646 polymorphism in studied subjects. (DOC) [file pone.0034828.s002.doc]

**Table S2: Genotypic and allelic distribution of *CYP19A1* rs4646 polymorphism** **in** **studied subjects**

|  | Genotypic distribution N(%) | | | Allelic distribution N(%) | |
| --- | --- | --- | --- | --- | --- |
|  | GG | GT | TT | G | T |
| Primary cohort | | | | | |
| Migraine(207) | 108(52.2) | 74(35.7) | 25(12.1) | 290(70.05) | 124(29.95) |
| MO(129) | 64(49.6) | 47(36.4) | 18(14.0) | 175(68.90) | 79(31.10) |
| MA(78) | 44(56.4) | 27(34.6) | 7(9.0) | 115(73.72) | 41(26.28) |
| Females |  |  |  |  |  |
| Migraine(141) | 75(53.2) | 50(35.5) | 16(11.3) | 200(70.92) | 82(29.08) |
| MO(84) | 40(47.6) | 32(38.1) | 12(14.3) | 112(66.67) | 56(33.33) |
| MA(57) | 35(61.4) | 18(31.6) | 4(7.0) | 88(77.19) | 26(22.81) |
| Males |  |  |  |  |  |
| Migraine(66) | 33(50.0) | 24(36.4) | 9(13.6) | 90(68.18) | 42(31.82) |
| MO(45) | 24(53.3) | 15(33.3) | 6(13.3) | 63(70.00) | 27(30.00) |
| MA(21) | 9(42.9) | 9(42.9) | 3(14.3) | 27(64.29) | 15(35.71) |
| Replicative cohort | | | | | |
| Migraine(127) | 72(56.7) | 35(27.6) | 20(15.7) | 179(70.47) | 75(29.53) |
| MO(99) | 53(53.5) | 28(28.3) | 18(18.2) | 134(67.68) | 64(32.32) |
| MA(28) | 19(67.9) | 7(25.0) | 2(7.1) | 45(80.36) | 11(19.64) |
| Females | | | | | |
| Migraine(93) | 53(57.0) | 25(26.9) | 15(16.1) | 131(70.43) | 55(29.57) |
| MO(72) | 39(54.2) | 20(27.8) | 13(18.1) | 98(68.06) | 46(31.94) |
| MA(21) | 14(66.7) | 5(23.8) | 2(9.5) | 33(78.57) | 9(21.43) |
| Males |  |  |  |  |  |
| Migraine(34) | 19(55.9) | 10(29.4) | 5(14.7) | 48(70.59) | 20(29.41) |
| MO(27) | 14(51.9) | 8(29.6) | 5(18.5) | 36(66.67) | 18(33.33) |
| MA(7) | 5(71.4) | 2(28.6) | 0(0) | 12(85.71) | 2(14.29) |
| Healthy controls | | | | | |
| HC(200) | 88(44.0) | 80(40.0) | 32(16.0) | 256(64.00) | 144(36.00) |
| Females(133) | 50(37.6) | 61(45.9) | 22(16.5) | 161(60.53) | 105(39.47) |
| Males(67) | 38(56.7) | 19(28.4) | 10(14.9) | 95(70.90) | 39(29.10) |
