# Supplementary material for: Potential Role of Aromatase over Estrogen Receptor Gene Polymorphisms in Migraine Susceptibility: A Case Control Study from North India
Source: PLoS One. 2012 Apr 12;7(4):e34828. doi: 10.1371/journal.pone.0034828 (PMC3325278; doi:10.1371/journal.pone.0034828)
Supplement: Table S3 — Genotypic and allelic distribution of ESR1 rs2234693 polymorphism in studied subjects. (DOC) [file pone.0034828.s003.doc]

**Table S3: Genotypic and allelic distribution of *ESR1* rs2234693 polymorphism** **in** **studied subjects**

|  | Genotypic distribution N(%) | | | Allelic distribution N(%) | |
| --- | --- | --- | --- | --- | --- |
|  | CC | CT | TT | C | T |
| Primary cohort | | | | | |
| Migraine(207) | 47(22.7) | 129(62.3) | 31(15.0) | 223(53.86) | 191(46.14) |
| MO(129) | 29(22.5) | 78(60.5) | 22(17.1) | 136(52.71) | 122(47.29) |
| MA(78) | 18(23.1) | 51(65.4) | 9(11.5) | 87(55.77) | 69(44.23) |
| Females |  |  |  |  |  |
| Migraine(141) | 33(23.4) | 90(63.8) | 18(12.8) | 156(55.32) | 126(44.68) |
| MO(84) | 20(23.8) | 52(61.9) | 12(14.3) | 92(54.76) | 76(45.24) |
| MA(57) | 13(22.8) | 38(66.7) | 6(10.5) | 64(56.14) | 50(43.86) |
| Males |  |  |  |  |  |
| Migraine(66) | 14(21.2) | 39(59.1) | 13(19.7) | 67(50.76) | 65(49.24) |
| MO(45) | 9(20.0) | 26(57.8) | 10(22.2) | 44(48.89) | 46(51.11) |
| MA(21) | 5(23.8) | 13(61.9) | 3(14.3) | 23(54.76) | 19(45.24) |
| Replicative cohort | | | | | |
| Migraine(127) | 49(38.6) | 54(42.5) | 24(18.9) | 152(59.84) | 102(40.16) |
| MO(99) | 43(43.4) | 41(41.4) | 15(15.2) | 127(64.14) | 71(35.86) |
| MA(28) | 6(21.4) | 13(46.4) | 9(32.1) | 25(44.64) | 31(55.36) |
| Females | | | | | |
| Migraine(93) | 39(41.9) | 36(38.7) | 18(19.4) | 114(61.29) | 72(38.71) |
| MO(72) | 33(45.8) | 27(37.5) | 12(16.7) | 93(64.58) | 51(35.42) |
| MA(21) | 6(28.6) | 9(42.9) | 6(28.6) | 21(50.00) | 21(50.00) |
| Males |  |  |  |  |  |
| Migraine(34) | 10(29.4) | 18(52.9) | 6(17.6) | 38(55.88) | 30(44.12) |
| MO(27) | 10(37.0) | 14(51.9) | 3(11.1) | 34(62.96) | 20(37.04) |
| MA(7) | 0(0.0) | 4(57.1) | 3(42.9) | 4(28.57) | 10(71.43) |
| Healthy controls | | | | | |
| HC(200) | 78(39.0) | 101(50.5) | 21(10.5) | 257(64.25) | 143(35.75) |
| Females(133) | 52(39.1) | 69(51.9) | 12(9.0) | 173(65.04) | 93(34.96) |
| Males(67) | 26(38.8) | 32(47.8) | 9(13.4) | 84(62.69) | 50(37.31) |
