# Supplementary material for: Potential Role of Aromatase over Estrogen Receptor Gene Polymorphisms in Migraine Susceptibility: A Case Control Study from North India
Source: PLoS One. 2012 Apr 12;7(4):e34828. doi: 10.1371/journal.pone.0034828 (PMC3325278; doi:10.1371/journal.pone.0034828)
Supplement: Table S4 — Genotypic and allelic distribution of ESR1 rs1801132 polymorphism in studied subjects. (DOC) [file pone.0034828.s004.doc]

**Table S4:Genotypic and allelic distribution of *ESR1* rs1801132 polymorphism** **in** **studied subjects**

|  | Genotypic distribution N(%) | | | Allelic distribution N(%) | |
| --- | --- | --- | --- | --- | --- |
|  | CC | CG | GG | C | G |
| Primary cohort | | | | | |
| Migraine(207) | 72(34.8) | 110(53.1) | 25(12.1) | 254(61.35) | 160(38.65) |
| MO(129) | 43(33.3) | 70(54.3) | 16(12.4) | 156(60.47) | 102(39.53) |
| MA(78) | 29(37.2) | 40(51.3) | 9(11.5) | 98(62.82) | 58(37.18) |
| Females |  |  |  |  |  |
| Migraine(141) | 51(36.2) | 72(51.1) | 18(12.8) | 174(61.70) | 108(38.30) |
| MO(84) | 28(33.3) | 44(52.4) | 12(14.3) | 100(59.52) | 68(40.48) |
| MA(57) | 23(40.4) | 28(49.1) | 6(10.5) | 74(64.91) | 40(35.09) |
| Males |  |  |  |  |  |
| Migraine(66) | 21(31.8) | 38(57.6) | 7(10.6) | 80(60.61) | 52(39.39) |
| MO(45) | 15(33.3) | 26(57.8) | 4(8.9) | 56(62.22) | 34(37.78) |
| MA(21) | 6(28.6) | 12(57.1) | 3(14.3) | 24(57.14) | 18(42.86) |
| Replicative cohort | | | | | |
| Migraine(127) | 62(48.8) | 56(44.1) | 9(7.1) | 180(70.87) | 74(29.13) |
| MO(99) | 47(47.5) | 45(45.5) | 7(7.1) | 139(70.20) | 59(29.80) |
| MA(28) | 15(53.6) | 11(39.3) | 2(7.1) | 41(73.21) | 15(26.79) |
| Females | | | | | |
| Migraine(93) | 45(48.4) | 40(43.0) | 8(8.6) | 130(69.89) | 56(30.11) |
| MO(72) | 34(47.2) | 31(43.1) | 7(9.7) | 99(68.75) | 45(31.25) |
| MA(21) | 11(52.4) | 9(42.9) | 1(4.8) | 31(73.81) | 11(26.19) |
| Males |  |  |  |  |  |
| Migraine(34) | 17(50.0) | 16(47.1) | 1(2.9) | 50(73.53) | 18(26.47) |
| MO(27) | 13(48.1) | 14(51.9) | 0(0.0) | 40(74.07) | 14(25.93) |
| MA(7) | 4(57.1) | 2(28.6) | 1(14.3) | 10(71.43) | 4(28.57) |
| Healthy controls | | | | | |
| HC(200) | 80(40.0) | 102(51.0) | 18(9.0) | 262(65.50) | 138(34.50) |
| Females(133) | 52(39.1) | 70(52.63) | 11(8.27) | 174(65.41) | 92(34.59) |
| Males(67) | 28(41.79) | 32(47.76) | 7(10.45) | 88(65.67) | 46(34.33) |
