# Supplementary material for: Potential Role of Aromatase over Estrogen Receptor Gene Polymorphisms in Migraine Susceptibility: A Case Control Study from North India
Source: PLoS One. 2012 Apr 12;7(4):e34828. doi: 10.1371/journal.pone.0034828 (PMC3325278; doi:10.1371/journal.pone.0034828)
Supplement: Table S5 — Genotypic and allelic distribution of ESR1 rs2228480 polymorphism in studied subjects. (DOC) [file pone.0034828.s005.doc]

**Table S5: Genotypic and allelic distribution of *ESR1* rs2228480 polymorphism in studied subjects**

|  | Genotypic distribution N(%) | | | Allelic distribution N(%) | |
| --- | --- | --- | --- | --- | --- |
|  | GG | GA | AA | G | A |
| Primary cohort | | | | | |
| Migraine(207) | 145(70.0) | 55(26.6) | 7(3.4) | 345(83.33) | 69(16.67) |
| MO(129) | 88(68.2) | 37(28.7) | 4(3.1) | 213(82.56) | 45(17.44) |
| MA(78) | 57(73.1) | 18(23.1) | 3(3.8) | 132(84.62) | 24(15.38) |
| Females |  |  |  |  |  |
| Migraine(141) | 94(66.7) | 41(29.1) | 6(4.3) | 229(81.21) | 53(18.79) |
| MO(84) | 55(65.5) | 26(31.0) | 3(3.6) | 136(80.95) | 32(19.05) |
| MA(57) | 39(68.4) | 15(26.3) | 3(5.3) | 93(81.58) | 21(18.42) |
| Males |  |  |  |  |  |
| Migraine(66) | 51(77.3) | 14(21.2) | 1(1.5) | 116(87.88) | 16(12.12) |
| MO(45) | 33(73.3) | 11(24.4) | 1(2.2) | 77(85.56) | 13(14.44) |
| MA(21) | 18(85.7) | 3(14.3) | 0(0) | 39(92.86) | 3(7.14) |
| Replicative cohort | | | | | |
| Migraine(127) | 81(63.8) | 41(32.3) | 5(3.9) | 203(79.92) | 51(20.08) |
| MO(99) | 65(65.7) | 30(30.3) | 4(4.0) | 160(80.81) | 38(19.19) |
| MA(28) | 16(57.1) | 11(39.3) | 1(3.6) | 43(76.79) | 13(23.21) |
| Females | | | | | |
| Migraine(93) | 63(67.7) | 26(28.0) | 4(4.3) | 152(81.72) | 34(18.28) |
| MO(72) | 49(68.1) | 20(27.8) | 3(4.2) | 118(81.94) | 26(18.06) |
| MA(21) | 14(66.7) | 6(28.6) | 1(4.8) | 34(80.95) | 8(19.05) |
| Males |  |  |  |  |  |
| Migraine(34) | 18(52.9) | 15(44.1) | 1(2.9) | 51(75.00) | 17(25.00) |
| MO(27) | 16(59.3) | 10(37.0) | 1(3.7) | 42(77.78) | 12(22.22) |
| MA(7) | 2(28.6) | 5(71.4) | 0(0.0) | 9(64.29) | 5(35.71) |
| Healthy controls | | | | | |
| HC(200) | 121(60.5) | 70(35.0) | 9(4.5) | 312(78.00) | 88(22.00) |
| Females(133) | 82(61.7) | 48(36.1) | 3(2.3) | 212(79.70) | 54(20.30) |
| Males(67) | 39(58.2) | 22(32.8) | 6(9.0) | 100(74.63) | 34(25.37) |
