# Supplementary material for: Potential Role of Aromatase over Estrogen Receptor Gene Polymorphisms in Migraine Susceptibility: A Case Control Study from North India
Source: PLoS One. 2012 Apr 12;7(4):e34828. doi: 10.1371/journal.pone.0034828 (PMC3325278; doi:10.1371/journal.pone.0034828)
Supplement: Table S6 — Genotypic and allelic distribution of ESR1 rs9340799 polymorphism in studied subjects. (DOC) [file pone.0034828.s006.doc]

**Table S6: Genotypic and allelic distribution of *ESR1* rs9340799 polymorphism** **in** **studied subjects**

|  | Genotypic distribution N(%) | | | Allelic distribution N(%) | |
| --- | --- | --- | --- | --- | --- |
|  | AA | AG | GG | A | G |
| Primary cohort | | | | | |
| Migraine(207) | 75(36.2) | 103(49.8) | 29(14.0) | 253(61.11) | 161(38.89) |
| MO(129) | 46(35.7) | 62(48.1) | 21(16.3) | 154(59.69) | 104(40.31) |
| MA(78) | 29(37.2) | 41(52.6) | 8(10.3) | 99(63.46) | 57(36.54) |
| Females |  |  |  |  |  |
| Migraine(141) | 50(35.5) | 71(50.4) | 20(14.2) | 171(60.64) | 111(39.36) |
| MO(84) | 30(35.7) | 41(48.8) | 13(15.5) | 101(55.49) | 81(44.51) |
| MA(57) | 20(35.1) | 30(52.6) | 7(12.3) | 70(61.40) | 44(38.60) |
| Males |  |  |  |  |  |
| Migraine(66) | 25(37.9) | 32(48.5) | 9(13.6) | 82(62.12) | 50(37.88) |
| MO(45) | 16(35.6) | 21(46.7) | 8(17.8) | 53(58.89) | 37(41.11) |
| MA(21) | 9(42.9) | 11(52.4) | 1(4.8) | 29(69.05) | 13(30.95) |
| Replicative cohort | | | | | |
| Migraine(127) | 50(39.4) | 57(44.9) | 20(15.7) | 157(61.81) | 97(38.19) |
| MO(99) | 44(44.4) | 44(44.4) | 11(11.1) | 132(66.67) | 66(33.33) |
| MA(28) | 6(21.4) | 13(46.4) | 9(32.1) | 25(44.64) | 31(55.36) |
| Females | | | | | |
| Migraine(93) | 39(41.9) | 38(40.9) | 16(17.2) | 116(62.37) | 70(37.63) |
| MO(72) | 33(45.8) | 29(40.3) | 10(13.9) | 95(65.97) | 49(34.03) |
| MA(21) | 6(28.6) | 9(42.9) | 6(28.6) | 21(50.00) | 21(50.0) |
| Males |  |  |  |  |  |
| Migraine(34) | 11(32.4) | 19(55.9) | 4(11.8) | 41(60.29) | 27(39.71) |
| MO(27) | 11(40.7) | 15(55.6) | 1(3.7) | 37(68.52) | 17(31.48) |
| MA(7) | 0(0.0) | 4(57.1) | 3(42.9) | 4(28.57) | 10(71.43) |
| Healthy controls | | | | | |
| HC(200) | 74(37.0) | 99(49.5) | 27(13.5) | 247(61.75) | 153(38.25) |
| Females(133) | 42(31.6) | 73(54.9) | 18(13.5) | 157(59.02) | 109(40.98) |
| Males(67) | 32(47.8) | 26(38.8) | 9(13.4) | 90(67.16) | 44(32.84) |
