# Supplementary material for: Potential Role of Aromatase over Estrogen Receptor Gene Polymorphisms in Migraine Susceptibility: A Case Control Study from North India
Source: PLoS One. 2012 Apr 12;7(4):e34828. doi: 10.1371/journal.pone.0034828 (PMC3325278; doi:10.1371/journal.pone.0034828)
Supplement: Table S7 — Genotypic and allelic distribution of ESR2 rs1271572 polymorphism in studied subjects. (DOC) [file pone.0034828.s007.doc]

**Table S 7: Genotypic and allelic distribution of *ESR2* rs1271572 polymorphism** **in** **studied subjects**

|  | Genotypic distribution N(%) | | | Allelic distribution N(%) | |
| --- | --- | --- | --- | --- | --- |
|  | AA | AC | CC | A | C |
| Primary cohort | | | | | |
| Migraine(207) | 106(51.2) | 87(42.0) | 14(6.8) | 299(72.22) | 115(27.78) |
| MO(129) | 65(50.4) | 55(42.6) | 9(7.0) | 185(71.71) | 73(28.29) |
| MA(78) | 41(52.6) | 32(41.0) | 5(6.4) | 114(73.08) | 42(26.92) |
| Females |  |  |  |  |  |
| Migraine(141) | 75(53.2) | 60(42.6) | 6(4.3) | 210(74.47) | 72(25.53) |
| MO(84) | 42(50.0) | 38(45.2) | 4(4.8) | 122(72.62) | 46(27.38) |
| MA(57) | 33(57.9) | 22(38.6) | 2(3.5) | 88(77.19) | 26(22.81) |
| Males |  |  |  |  |  |
| Migraine(66) | 31(47.0) | 27(40.9) | 8(12.1) | 89(67.42) | 43(32.58) |
| MO(45) | 23(51.1) | 17(37.8) | 5(11.1) | 63(70.00) | 27(30.00) |
| MA(21) | 8(38.1) | 10(47.6) | 3(14.3) | 26(61.90) | 16(38.10) |
| Replicative cohort | | | | | |
| Migraine(127) | 55(43.3) | 67(52.8) | 5(3.9) | 177(69.69) | 77(30.31) |
| MO(99) | 39(39.4) | 57(57.6) | 3(3.0) | 135(68.18) | 63(68.18) |
| MA(28) | 16(57.1) | 10(35.7) | 2(7.1) | 42(75.00) | 14(25.00) |
| Females | | | | | |
| Migraine(93) | 38(40.9) | 50(53.8) | 5(5.4) | 126(67.74) | 60(32.26) |
| MO(72) | 28(38.9) | 41(56.9) | 3(4.2) | 97(67.36) | 47(32.64) |
| MA(21) | 10(47.6) | 9(42.9) | 2(9.5) | 29(69.05) | 13(30.95) |
| Males |  |  |  |  |  |
| Migraine(34) | 17(50.0) | 17(50.0) | 0(0) | 51(75.00) | 17(25.00) |
| MO(27) | 11(40.7) | 16(59.3) | 0(0) | 38(70.37) | 16(29.63) |
| MA(7) | 6(85.7) | 1(14.3) | 0(0) | 13(92.86) | 1(7.14) |
| Healthy controls | | | | | |
| HC(200) | 89 (44.5) | 95(47.5) | 16(8.0) | 273(68.25) | 127(31.75) |
| Females(133) | 66(49.6) | 57(42.9) | 10(7.5) | 189(71.05) | 77(28.95) |
| Males(67) | 23(34.3) | 38(56.7) | 6(9.0) | 84(62.69) | 50(37.31) |
