# Supplementary material for: Potential Role of Aromatase over Estrogen Receptor Gene Polymorphisms in Migraine Susceptibility: A Case Control Study from North India
Source: PLoS One. 2012 Apr 12;7(4):e34828. doi: 10.1371/journal.pone.0034828 (PMC3325278; doi:10.1371/journal.pone.0034828)
Supplement: Table S8 — Genotypic and allelic distribution of ESR2 rs1256049 polymorphism in studied subjects. (DOC) [file pone.0034828.s008.doc]

**Table S 8: Genotypic and allelic distribution of *ESR2* rs1256049 polymorphism** **in** **studied subjects**

|  | Genotypic distribution N(%) | | | Allelic distribution N(%) | |
| --- | --- | --- | --- | --- | --- |
|  | GG | GA | AA | G | A |
| Primary cohort | | | | | |
| Migraine(207) | 192 (92.8) | 15(7.2) | 0(0) | 399(96.38) | 15(3.62) |
| MO(129) | 121(93.8) | 8(6.2) | 0(0) | 250(96.90) | 8(3.10) |
| MA(78) | 71(91.0) | 7(9.0) | 0(0) | 149(95.51) | 7(4.49) |
| Females |  |  |  |  |  |
| Migraine(141) | 131(92.9) | 10(7.1) | 0(0) | 272(96.45) | 10(3.55) |
| MO(84) | 79(94.0) | 5(6.0) | 0(0) | 163(97.02) | 5(2.98) |
| MA(57) | 52(91.2) | 5(8.8) | 0(0) | 109(95.61) | 5(4.39) |
| Males |  |  |  |  |  |
| Migraine(66) | 61(92.4) | 5(7.6) | 0(0) | 127(96.21) | 5(3.79) |
| MO(45) | 42(93.3) | 3(6.7) | 0(0) | 87(96.67) | 3(3.33) |
| MA(21) | 19(90.5) | 2(9.5) | 0(0) | 40(95.24) | 2(4.76) |
| Replicative cohort | | | | | |
| Migraine(127) | 116(91.3) | 11(8.7) | 0(0) | 243(95.67) | 11(4.33) |
| MO(99) | 91(91.9) | 8(8.1) | 0(0) | 190(95.96) | 8(4.04) |
| MA(28) | 25(89.3) | 3(10.7) | 0(0) | 53(94.64) | 3(5.36) |
| Females | | | | | |
| Migraine(93) | 85(91.4) | 8(8.6) | 0(0) | 178(95.70) | 8(4.30) |
| MO(72) | 66(91.7) | 6(8.3) | 0(0) | 138(95.83) | 6(4.17) |
| MA(21) | 19(90.5) | 2(9.5) | 0(0) | 40(95.24) | 2(4.76) |
| Males |  |  |  |  |  |
| Migraine(34) | 31(91.2) | 3(8.8) | 0(0) | 65(95.59) | 3(4.41) |
| MO(27) | 25(92.6) | 2(7.4) | 0(0) | 52(96.30) | 2(3.70) |
| MA(7) | 6(85.7) | 1(14.3) | 0(0) | 13(92.86) | 1(7.14) |
| Healthy controls | | | | | |
| HC(200) | 193(96.5) | 7(3.5) | 0(0.0) | 393(98.25) | 7(1.75) |
| Females(133) | 128(96.2) | 5(3.8) | 0(0.0) | 261(98.12) | 5(1.88) |
| Males(67) | 65(97.0) | 2(3.0) | 0(0.0) | 132(98.51) | 2(1.49) |
