# Supplementary material for: Potential Role of Aromatase over Estrogen Receptor Gene Polymorphisms in Migraine Susceptibility: A Case Control Study from North India
Source: PLoS One. 2012 Apr 12;7(4):e34828. doi: 10.1371/journal.pone.0034828 (PMC3325278; doi:10.1371/journal.pone.0034828)
Supplement: Table S9 — Haplotype analysis of ESR1 gene polymorphisms. (DOC) [file pone.0034828.s009.doc]

**Table S9: Haplotype analysis of *ESR1* gene polymorphisms**

| **Haplotype^a^**  **(rs2234693-rs1801132- rs2228480- rs9340799)** | **Name** | **Healthy Controls**  **(n=200)** | **Primary cohort**  **(n=207)** | | | **Replicative cohort**  **(n=127)** | | | **Meta Analysis** | | | |
| --- | --- | --- | --- | --- | --- | --- | --- | --- | --- | --- | --- | --- |
|  |  |  |  |  |  |  |  |  | **Fisher’s method** | | **Benjamini-Hochberg FDR test** | **Mantel-Haenszel test** |
|  |  | **N(%)** | **N(%)** | **p ^b^** | **OR(95%CI)** | **N(%)** | **p ^b^** | **OR(95%CI)** | **Χ^2^** | **p ^b^** | **p _corr_ ^b^** | **OR_MH_(95%CI)** |
| 0000 | A | 47(23.5) | 50(24.2) | Reference | | 38(29.9) | Reference | | - | - | - | - |
| 0001 | B | 13(6.5) | 2(0.96) | **.014** | .145(0.031-0.675) | 1(.8) | **.027** | .095(0.012-0.760) | 15.76 | **0.0034** | **0.01** | 0.129(0.03828-0.4347) |
| 0010 | C | 20(10.0) | 11(5.3) | .122 | .517 (0.224-1.193) | 9(7.1) | .200 | .557(0.227-1.363) | - | - | - | - |
| 0100 | D | 36(18.0) | 34(16.4) | .704 | .888(0.480-1.642) | 21(16.5) | .352 | .721(0.363-1.435) | - | - | - | - |
| 1000 | E | 10(5.0) | 11(5.3) | .945 | 1.034(0.402-2.659) | 3(2.4) | .153 | .371(0.095-1.445) | - | - | - | - |
| 0101 | F | 3(1.5) | 2(1.0) | .617 | .627(0.100-3.918) | 1(0.8) | .451 | .412(0.041-4.125) | - | - | - | - |
| 0110 | G | 4(2.0) | 11(5.3) | .124 | 2.585(0.770-8.683) | 5(3.9) | .537 | 1.546(0.388-6.161) | - | - | - | - |
| 1001 | H | 32(16.0) | 45(21.7) | .365 | 1.322(0.723-2.417) | 30(23.6) | .659 | 1.160(0.601-2.236) | - | - | - | - |
| 1010 | I | 4(2.0) | 2(1.0) | .396 | .470(0.082-2.687) | 2(1.6) | .590 | .618(0.107-3.560) | - | - | - | - |
| 1100 | J | 2(1.0) | 4(1.9) | .478 | 1.880(0.329-10.748) | 1(0.8) | .699 | .618(0.054-7.083) | - | - | - | - |
| 0011 | K | 2(1.0) | 1(0.5) | .543 | .470(0.041-5.356) | 1(0.8) | .699 | .618(0.054-7.083) | - | - | - | - |
| 1011 | L | 4(2.0) | 4(1.9) | .933 | .940(0.222-3.975) | 6(4.7) | .364 | 1.855(0.488-7.053) | - | - | - | - |
| 1101 | M | 12(6.0) | 24(11.6) | .122 | 1.880(0.845-4.181) | 6(4.7) | .378 | .618(0.212-1.801) | - | - | - | - |
| 1110 | N | 1(0.5) | 3(1.4) | .377 | 2.820(0.283-28.069) | 0 | - | - | - | - | - | - |
| 0111 | O | 4(2.0) | 1(0.5) | .203 | .235(0.025-2.179) | 0 | - | - | - | - | - | - |
| 1111 | P | 6(3.0) | 2(1.0) | .168 | .313(0.060-1.630) | 3(2.4) | .516 | .618(0.145-2.637) | - | - | - | - |

OR odds ratio, CI confidence interval, OR_MH_ Mantel – Heanszel odds ratio

^a^0&1 represent wild and variant, respectively

^b^ p Values in bold denotes significance
