# Supplementary material for: Potential Role of Aromatase over Estrogen Receptor Gene Polymorphisms in Migraine Susceptibility: A Case Control Study from North India
Source: PLoS One. 2012 Apr 12;7(4):e34828. doi: 10.1371/journal.pone.0034828 (PMC3325278; doi:10.1371/journal.pone.0034828)
Supplement: Table S10 — Association study of ESR2 polymorphisms. (DOC) [file pone.0034828.s010.doc]

**Table S10 : Association study of *ESR2*** polymorphisms

|  | **Primary cohort** | | **Replicative cohort** | | **Meta analysis** | | |
| --- | --- | --- | --- | --- | --- | --- | --- |
|  |  |  |  |  | **Fisher’s method** | | **Mantel-Haenszel test** |
|  | **p** | **OR(95%CI)** | **p** | **OR(95%CI)** | **Χ2** | **p** | **OR_MH_(95%CI)** |
| **ESR2 rs1271572** | | | | | | | |
| **Association of CC genotype with migraine susceptibility** | | | | | | | |
| Migraine Vs HC | 0.406 | 0.719(0.330-1.566) | 0.171 | 0.474(0.163-1.379) | - | - | - |
| MO Vs HC | 0.518 | 0.747(0.309-1.808) | 0.172 | 0.404(0.110-1.483) | - | - | - |
| MA Vs HC | 0.453 | 0.659(0.222-1.958) | 0.596 | 0.651(0.134-3.172) | - | - | - |
| **ESR2 rs 1256049** | | | | | | | |
| **Association of GA genotype with migraine susceptibility** | | | | | | | |
| Migraine Vs HC | 0.110 | 2.121(0.845-5.328) | 0.064 | 2.522(0.946-6.721) | - | - | - |
| MO Vs HC | 0.266 | 1.805(0.637-5.111) | 0.116 | 2.322(0.813-6.632) | - | - | - |
| MA Vs HC | 0.088 | 2.585(0.868-7.694) | 0.120 | 3.118(0.744-13.067) | - | - | - |

MA migraine with aura, MO migraine without aura, HC healthy controls, OR odds ratio, CI confidence interval, OR_MH_ Mantel – Heanszel odds ratio
